# A spatiotemporal model of spine calcium dynamics in the hippocampus

**DOI:** 10.1186/1471-2202-16-S1-P268

**Published:** 2015-12-18

**Authors:** Thom Griffith, Jack Mellor, Krasi Tsaneva-Atanasova

**Affiliations:** 1 Department of Engineering Maths, University of Bristol, Bristol, UK; 2 School of Physiology and Pharmacology, University of Bristol, Bristol, UK; 3 Department of Mathematics, University of Exeter, Exeter, UK

## 

Ca^2+^-signalling in dendritic spines is required for NMDA receptor-dependent synaptic plasticity at glutamatergic synapses in the hippocampus [1]. However, it is not clear whether plasticity induction is dependent solely on the global signal, i.e., the spine volume-averaged Ca^2+ ^signal; or whether plasticity induction is also sensitive to Ca^2+^-channel nanodomain signaling [2]. A working hypothesis of this work is that temporal and spatial variations in postsynaptic intracellular [Ca^2+^]-fields may be significant factors governing the signalling cascades that lead to either long-term synaptic potentiation or depression. Direct measurement of [Ca^2+^] distributions in dendritic spines is experimentally difficult but we can investigate this hypothesis using mathematical models of Ca^2+ ^diffusion.

We have developed a spatio-temporal model of Ca^2+ ^diffusion in three dimensions. We then study our model using finite element methods. The model allows predictions of intracellular [Ca^2+^]-field responses to combinations of pre- and post-synaptic spikes with nanometre and millisecond spatio-temporal resolution. Our results so far indicate that Ca^2+ ^signalling is highly spatially non-uniform and that Ca^2+ ^signal differences between induction protocols is dependent on location within the spine. This has implications for the ultimate biological role of the Ca^2+ ^signal given that the relevant receptors in the spine are organised inhomogeneously [3].
